# Pan-tissue transcriptome analysis of long noncoding RNAs in the American beaver *Castor canadensis*

**DOI:** 10.1186/s12864-019-6432-4

**Published:** 2020-02-12

**Authors:** Amita Kashyap, Adelaide Rhodes, Brent Kronmiller, Josie Berger, Ashley Champagne, Edward W. Davis, Mitchell V. Finnegan, Matthew Geniza, David A. Hendrix, Christiane V. Löhr, Vanessa M. Petro, Thomas J. Sharpton, Jackson Wells, Clinton W. Epps, Pankaj Jaiswal, Brett M. Tyler, Stephen A. Ramsey

**Affiliations:** 1 Department of Biomedical Sciences, Oregon State University, Corvallis, OR USA; 2 Center for Genome Research and Biocomputing, Oregon State University, Corvallis, OR USA; 3 College of Forestry, Oregon State University, Corvallis, OR USA; 4 Department of Fisheries and Wildlife, Oregon State University, Corvallis, OR USA; 5 Oregon Zoo, Portland, OR USA; 6 Department of Botany and Plant Pathology, Oregon State University, Corvallis, OR USA; 7 Department of Biochemistry and Biophysics, Oregon State University, Corvallis, OR USA; 8 School of Electrical Engineering and Computer Science, Oregon State University, Corvallis, OR USA; 9 Department of Microbiology, Oregon State University, Corvallis, OR USA; 10 Department of Statistics, Oregon State University, Corvallis, OR USA

**Keywords:** lncRNA, Beaver, Transcriptome, Long noncoding RNA, *Castor canadensis*, Expression atlas

## Abstract

**Background:**

Long noncoding RNAs (lncRNAs) have roles in gene regulation, epigenetics, and molecular scaffolding and it is hypothesized that they underlie some mammalian evolutionary adaptations. However, for many mammalian species, the absence of a genome assembly precludes the comprehensive identification of lncRNAs. The genome of the American beaver (*Castor canadensis*) has recently been sequenced, setting the stage for the systematic identification of beaver lncRNAs and the characterization of their expression in various tissues. The objective of this study was to discover and profile polyadenylated lncRNAs in the beaver using high-throughput short-read sequencing of RNA from sixteen beaver tissues and to annotate the resulting lncRNAs based on their potential for orthology with known lncRNAs in other species.

**Results:**

Using de novo transcriptome assembly, we found 9528 potential lncRNA contigs and 187 high-confidence lncRNA contigs. Of the high-confidence lncRNA contigs, 147 have no known orthologs (and thus are putative novel lncRNAs) and 40 have mammalian orthologs. The novel lncRNAs mapped to the Oregon State University (OSU) reference beaver genome with greater than 90% sequence identity. While the novel lncRNAs were on average shorter than their annotated counterparts, they were similar to the annotated lncRNAs in terms of the relationships between contig length and minimum free energy (MFE) and between coverage and contig length. We identified beaver orthologs of known lncRNAs such as *XIST*, *MEG3*, *TINCR*, and *NIPBL-DT.* We profiled the expression of the 187 high-confidence lncRNAs across 16 beaver tissues (whole blood, brain, lung, liver, heart, stomach, intestine, skeletal muscle, kidney, spleen, ovary, placenta, castor gland, tail, toe-webbing, and tongue) and identified both tissue-specific and ubiquitous lncRNAs.

**Conclusions:**

To our knowledge this is the first report of systematic identification of lncRNAs and their expression atlas in beaver. LncRNAs—both novel and those with known orthologs—are expressed in each of the beaver tissues that we analyzed. For some beaver lncRNAs with known orthologs, the tissue-specific expression patterns were phylogenetically conserved. The lncRNA sequence data files and raw sequence files are available via the web supplement and the NCBI Sequence Read Archive, respectively.

## Background

Long noncoding RNAs (lncRNAs)—functional ribonucleic acids that do not encode proteins and are at least 200 nucleotides (nt) in length [1]—regulate gene expression through diverse mechanisms including epigenetic, chromatin, and molecular scaffolding interactions. For example, the primary effector for X-chromosome inactivation, *XIST*, is a lncRNA [2]. More broadly, various noncoding RNAs (ncRNAs) have been implicated in host defense against specific pathogens and in responses to various stressors, including hypoxia [3, 4]. Mounting evidence implicating species-specific ncRNAs and gene regulatory mechanisms in species adaptations [3, 5], including various species-specific responses to hypoxia [3, 4], suggests that species-specific and taxon-specific lncRNAs may underlie some of the adaptations seen in mammalian evolution. However, out of more than five thousand extant mammalian species (estimated as of 2019), less than 90 have high-quality genome assemblies available (according to the Ensembl genome database [6] release 96), and for those that do not, the absence of a genome or transcriptome sequence precludes comprehensive sequencing-based identification of lncRNAs.

The genome and three tissue transcriptomes of the American beaver *Castor canadensis* (Order Rodentia, Family Castoridae) have recently been sequenced [7, 8], enabling the systematic search for molecular determinants of this semi-aquatic herbivore’s unique physiologic, anatomic, and behavioral adaptations. For example, the beaver’s ability to hold its breath for up to fifteen minutes [9] suggests adaptations in the brain, heart, liver, and lungs to mitigate hypoxia-associated tissue damage and optimize oxygen uptake [10]. The beaver’s abilities to digest tree bark [11] and certain toxic plants [12] may depend on adaptations of detoxifying enzymes [13, 14] and lignocellulose-catabolizing gut microbes [15]. Such enzymatic adaptations may involve novel lncRNAs. Indeed, lncRNAs have been implicated in species-specific adaptations such as hibernation in grizzly bears [16] and adaptation to cold in zebrafish [17]. Therefore, establishing a compendium of beaver lncRNAs (both novel lncRNAs and those that are orthologous to known lncRNAs in other species) is an important starting point for efforts to understand the roles of noncoding RNAs in regulating expression of genes that underlie beaver anatomy and physiology.

Current high-throughput approaches for transcriptome profiling—especially for species for which only a draft reference genome is available—typically produce a fragmented transcriptome [18]. As a result, in the absence of an annotated genome, delineating a lncRNA transcript from a noncoding portion of a protein-coding transcript poses a bioinformatics challenge. Because a lncRNA is defined by *not* encoding a protein product, it is not possible to definitively identify a potential lncRNA by isolating a novel protein product, as is the case with an mRNA. Furthermore, lncRNAs often have weak sequence similarity across species [19], and the catalogue of validated lncRNAs outside of model vertebrates (human, mouse, rat) is incomplete. However, computational tools are now available for accurately scoring a transcript’s coding potential based on its sequence (e.g., longest ORF and hexamer usage bias [20]), closing a key informatics gap for lncRNA discovery.

We report on the first effort (of which we are aware) to systematically identify and map polyadenylated lncRNAs in the American beaver. Our rationale for focusing on polyadenylated lncRNAs (vs. non-polyadenylated lncRNAs) is twofold: (1) biologically, the majority of functional lncRNAs reported to date are polyadenylated [21] and polyadenylated lncRNAs in general are expressed at higher abundances than non-polyadenylated lncRNAs [22]; and (2) from a technical standpoint, use of poly-A selection enables strand-specific transcript profiling and avoids the requirement to validate (and ascertain the biases introduced by) the use of ribosomal RNA (rRNA) probe reagents in a species for which the reagents have not previously been tested [23]. As the foundation for this effort, we used the recently-released Oregon State University beaver genome assembly (see Methods) and we acquired and analyzed high-throughput, short-read polyadenylated RNA sequence data from 16 beaver tissues. We designed and implemented a computational analysis software pipeline for (1) assembling a pan-tissue beaver transcriptome; (2) identifying candidate lncRNA contigs based on evidence for coding potential and annotations of orthologous genes; and (3) measuring expression levels of the lncRNA contigs in the 16-tissue atlas. We identified 9528 potential lncRNA contigs which we then more stringently filtered by computational assessment of coding potential in order to minimize the number of coding transcripts erroneously identified as lncRNAs. We thus identified 187 putative lncRNAs in the beaver transcriptome, of which 147 appear to be novel and 40 are orthologs of known noncoding transcripts in other species, such as *XIST*, *MEG3*, *TINCR*, and *NIPBL-DT*. From the measured expression levels of the 187 lncRNAs across the 16 tissues, we (i) identified both tissue-specific and tissue-ubiquitous lncRNAs, (ii) correlated tissue expression profiles of three beaver lncRNAs with the tissue expression profiles of their orthologs and (iii) identified biological pathways and biological processes that beaver lncRNAs may regulate. These results lay the groundwork for studying the cellular and biochemical mechanisms underlying the beaver’s unique physiology and provide an analysis approach that can be used in lncRNA studies in other species.

## Results

### Screening pipeline

In order to obtain a comprehensive profile of the noncoding transcriptome of the American beaver, we paired-end sequenced polyadenylated RNA pooled from samples of sixteen different beaver tissues and de novo assembled a “pan-tissue” beaver polyadenylated RNA transcriptome using Trinity (see Methods). We merged the transcript contigs into 86,714 non-redundant contigs which became the basis for the remainder of the lncRNA screen. As a test of the completeness of the pan-tissue beaver polyadenylated RNA transcriptome, we used a benchmark set of 4014 genes (the mammalian Benchmarking Universal Single-Copy Ortholog [BUSCO] genes; see Methods) that had been previously validated as universal single-copy orthologs across various genome-sequenced mammalian species [24]. We found that 66% of the mammalian BUSCO genes had high-confidence (*E* <  10^− 5^) matches to one or more contigs in the Trinity-assembled, pan-tissue, beaver polyadenylated RNA transcriptome.

We filtered the 86,714 pan-tissue beaver transcript contigs to identify probable lncRNA contigs using five filtering steps, each shown in a row of Table 1: (1) identifying transcript contigs that have annotated orthologs in other species; this included identifying contigs with lncRNA orthologs (“known lncRNAs”, which were further curated); (2) filtering based on contigs’ coding potential score (*p* ≤ 0.01) as predicted based on their hexamer sequence content and the length of and coverage of the transcript by the longest Open Reading Frame (ORF); (3) more stringently filtering based on contigs’ Coding Potential Assessment Tool (CPAT) score (*q* ≤ 0.01; see Methods) to obtain a set of high-confidence noncoding contigs; (4) testing contigs for known protein domain sequences; and (5) aligning to the annotated reference beaver genome assembly, to determine if a transcript contig was in an untranslated region of a protein-coding gene. At Step 2, we obtained 9528 probable-noncoding contigs (see Additional file 3 Supplementary Data 1 for sequences). With a more stringent cutoff to control for false discovery rate (Step 3), and including additional filtering steps (4) and (5), we found a total of 187 probable lncRNA contigs: 40 noncoding transcript contigs that are orthologous to a known noncoding transcript in another species such as human or mouse (“known lncRNAs”) and 147 noncoding transcript contigs (see Table 1, bottom row) that appear to be novel from a species orthology standpoint (“novel lncRNAs”) (see Additional file 4 Supplementary Data 2 for sequences).

**Table 1:** Contig retention through the screening pipeline for novel lncRNAs

Step	% Contigs Eliminated	# Contigs Eliminated	# Contigs Remaining
Orthology analysis (BLASTn)	62.7	54,405 (^a^)	32,309 novel
Probable noncoding (CPAT *p* < 0.01)	70.1	22,781	9528
High confidence noncoding (CPAT *q* < 0.01)	98.1	9346	182
Pfam annotations	0	0	182
align to genome and compare to MAKER annotations	19.2	35	147

Columns as follows: “Step”, the name of the program or step in the screening pipeline; “% Contigs Eliminated”, the percentage of contigs from Column 4 of the previous row in the table that were eliminated in this step of the analysis pipeline; “# Contigs Eliminated”, the number of contigs corresponding to the percentage in Column 2; “# Contigs Remaining”, the number of contigs remaining after the row’s filtering Step was applied. The number of starting contigs before step 1 (“Orthology analysis”) was 86,714

(^a^) This includes the 40 beaver contigs that we identified that are orthologs of known noncoding transcripts in other species (Fig. 9, purple rectangle). The percentage shown in column “% Contigs Eliminated” is for that specific step (row) relative to the number of contigs before that step.

### Length and secondary structure characterization of known and novel lncRNA contigs

**Fig. 1:**
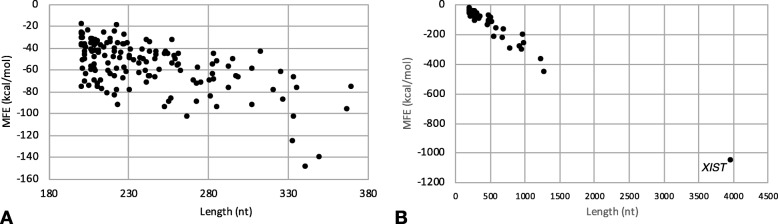
Noncoding transcript contigs’ model-based structural stability is inversely correlated with length. Marks indicate lncRNA contigs that have no known orthologs (“novel”; **a**) and that have known noncoding orthologs (“known”, **b**). The outlier in (**b**) is labeled by its known ortholog, *XIST*

To the extent that lncRNA biological function depends on a sufficiently stable structural conformation [25], in order to quantitatively assess the noncoding contigs’ potential for function, we computationally modeled the secondary structures and obtained model-based Minimum Free Energy (MFE) estimates for all 187 (known and novel) contigs (see Methods). Both sets of lncRNAs had the expected inverse relationship between transcript (contig) length and MFE, though the relationship was weaker in the novel lncRNAs (Fig. 1).

**Fig. 2:**
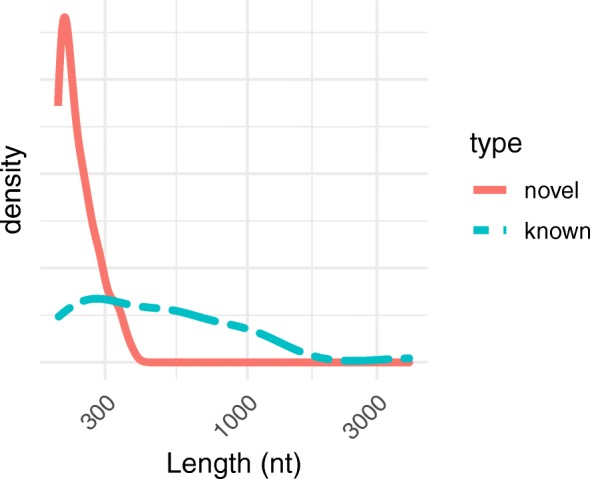
The lncRNA contigs with known orthologs are longer than the novel lncRNA contigs. Density distributions of contig lengths for the 147 novel noncoding transcript contigs (“novel”) and the 40 noncoding transcript contigs that are orthologous to known noncoding transcripts (“known”)

Overall, the transcript contigs for known lncRNAs were significantly (*p* <  10^− 9^; Kolmogorov-Smirnov test) longer than those of the novel lncRNAs (Fig. 2). Whereas the annotated lncRNAs were in the range of 204–4691 nt in length (consistent with GENCODE [26]), the putative novel lncRNA contigs were all below 400 nt in length. This is consistent with previous RNA-seq-based lncRNA studies which have tended to produce shorter contigs (less than 400 nt) even with genome-guided assembly [27, 28].

**Fig. 3:**
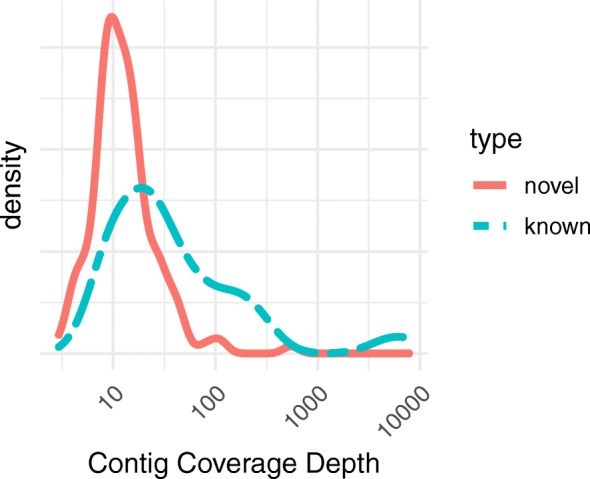
In the pan-tissue transcriptome assembly, known lncRNA contigs had overall higher coverage levels than novel lncRNA contigs. Density distributions of contig coverage depths for the 147 novel noncoding transcript contigs (“novel”) and the 40 noncoding transcript contigs that are orthologous to known noncoding transcripts (“known”). For both sets of noncoding transcript contigs, average depth of coverage in the assembly was not significantly correlated with contig length (Fig. 5)

In terms of read-depth coverage level in the transcriptome assembly, the distributions for the two sets of noncoding transcript contigs were both right-skewed (Fig. 3). Contigs with orthologs that are known noncoding transcripts (“known”) had higher average coverage depth (mode of 20.0, average of 369) than the noncoding transcript contigs with no known orthologs (“novel”; mode of 9.5, average of 19.4); the difference between the sets of contigs was not as striking for coverage as for length.

### The putative novel lncRNAs map back to the draft beaver genome

As a quality check, we aligned the 147 novel noncoding contigs to a reference beaver genome assembly (Oregon State University beaver genome assembly; see Methods). Every transcript contig aligned with upwards of 90% identity, and over 91% of putative novel lncRNA contigs had an alignment equivalent to at least 70% of the contig’s length (Additional file 1 Figure. S1). One contig (Ccan_OSU1_lncRNA_contig62060.1) had two non-overlapping alignments within 33 nucleotides of each other on the draft genome, which may indicate excision of an intron. To further validate the 147 novel contigs, we aligned them against a completely independently-generated beaver genome assembly [7] using BLASTn (see Methods); 144 of them (all except contig72949.1, contig80019.1, and contig83657.1) aligned with a best-match *E-*value of less than 10^− 18^. Of the 144 aligned contigs, all of them had greater than 90% sequence mapped and 140 of them had greater than 95% sequence mapped.

### Novel lncRNAs in the American beaver

**Fig. 4:**
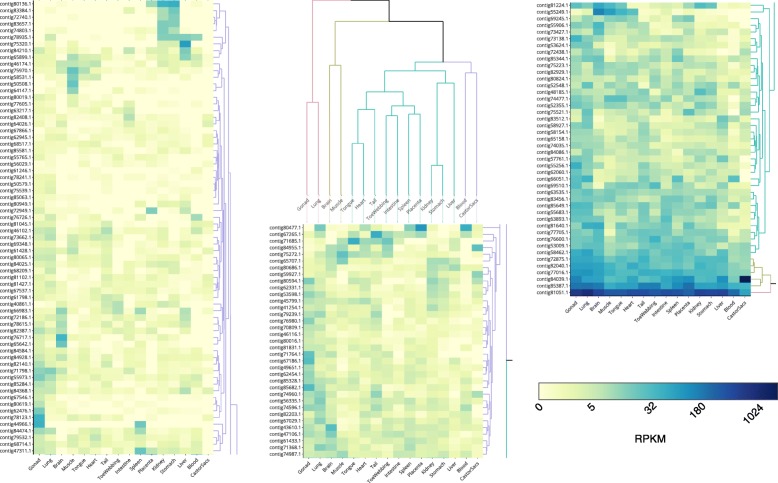
Tissue-specific expression of novel lncRNAs in the American beaver. Heatmap rows correspond to the 147 contigs and columns correspond to the 16 tissues that were profiled. Cells are colored by log_2_(1 + RPKM) expression level. Rows and columns are separately ordered by hierarchical agglomerative clustering and cut-based sub-dendrograms are colored (arbitrary color assignment to sub-clusters) as a guide for visualization. Rows are labeled with abbreviated contig names, e.g., contig4731.1 instead of Ccan_OSU1_lncRNA_contig4731.1

The novel lncRNAs as a group performed similarly to their annotated counterparts on the measures that we used to determine biological plausibility. Eight candidate lncRNAs stood out, however, for having the strongest evidence across the various measures (Table 2). Five of these contigs were among the top ten contigs in terms of at least length and MFE. This concordance between length and MFE is not surprising in light of the inverse relationship between transcript length and secondary structural stability (Fig. 1). One novel lncRNA (Ccan_OSU1_lncRNA_contig62060.1) was notable for having two exons, as detected by gapped alignment to the beaver genome. All of the eight novel contigs had robust expression (⩾ 6.5) in at least one tissue, as measured by Reads Per Kilobase of transcript per Million (RPKM) (see Table 2; Fig. 4; Methods).

**Table 2:** Novel lncRNA contigs with strongest evidence across multiple correlates

Contig	Measure					max (RPKM)
	Length (nt)	MFE (kcal/mol)	Coverage	BLASTn Alignment Length (%)	Intronic	
Ccan_OSU1_lncRNA_contig41254.1	367	−96.8	26.71	100.00	no	7.8
Ccan_OSU1_lncRNA_contig46102.1	334	− 103.57	8.42	100.00	no	7.6
Ccan_OSU1_lncRNA_contig46174.1	333	− 126.5	16.66	100.00	no	6.5
Ccan_OSU1_lncRNA_contig43610.1	350	−140.8	10.21	83.71	no	30.1
Ccan_OSU1_lncRNA_contig44966.1	341	− 149.8	11.81	63.93	no	48.6
Ccan_OSU1_lncRNA_contig45799.1	336	− 77	16.06	100.00	no	8.0
Ccan_OSU1_lncRNA_contig59927.1	267	−103.7	13.66	100.75	no	13.0
Ccan_OSU1_lncRNA_contig62060.1	260	−50.7	36.25	69.23	yes	22.8

Underlined text indicates that a particular contig was in the top ten, among all novel lncRNA contigs, for the given column feature (i.e., length, MFE, coverage, or alignment length). The BLASTn alignment length is computed as 100×(length of alignment)/(length of contig). The sixth column (Intronic) reflects whether the contig’s alignment to the reference genome was gapped or not; a “yes” is indicative of a potential excised intron. The last column, max (RPKM), is the maximum RPKM for the contig across all tissues and was not a criteria for inclusion in the table

**Fig. 5:**
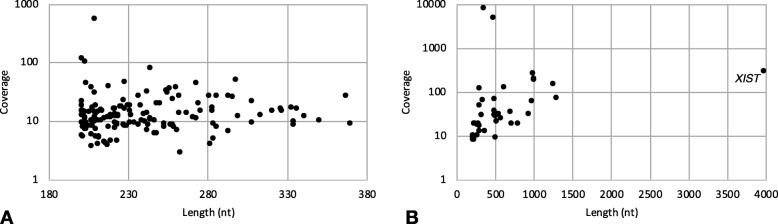
Contig average depth of read coverage in the assembly is not correlated with contig length. Marks indicate contigs that do not have orthologs (**a**, 147 contigs) or that are orthologous to known noncoding transcripts (**b**, 40 contigs). The outlier in (**b**) is labeled by its known ortholog, *XIST*

Interestingly, none of the eight lncRNAs were among those contigs with the highest coverage. This may be explained by the weakness of the relationship between length and observed coverage of novel lncRNA transcripts (Fig. 5). Furthermore, among the novel transcripts, the four contigs with exceptionally high coverage had coverage that was, on average, 15-fold greater than that of the rest of the contigs. Additionally, all of these contigs with exceptionally high coverage were under 250 nt long, while the ten longest novel lncRNAs were over 300 nt.

### Beaver orthologs of known lncRNAs or known noncoding transcript isoforms

Of the 40 lncRNA contigs for which a high-confidence ortholog gene could be identified, the ortholog annotations included 16 long noncoding RNA genes, 12 noncoding antisense RNAs, ten noncoding isoforms of protein-coding genes, and two sense-overlapping RNAs (Table 3). The relatively large proportion (12 out of 40) of antisense RNAs is consistent with a previous report that antisense transcripts are highly prevalent in the human genome [29]. The list of 16 lncRNA genes includes beaver orthologs for well-known lncRNAs such as *XIST* [2] (which was the longest of 187 high-confidence lncRNA contigs at 3967 nt), maternally expressed gene 3 (*MEG3*) [30], terminal differentiation-induced non-coding RNA (*TINCR*) [31], and nipped-B homolog (*Drosophila*) long noncoding RNA bidirectional promoter (*NIPBL-DT*) [32].

**Table 3:** Beaver noncoding contigs that are probable orthologs of known lncRNAs or noncoding transcripts

Symbol; annotation	Contig	Species with ortholog hits	Human Ensembl Gene ID	BLASTn annotation	*E*	%ID	nt
AC037459.2; (antisense to CCAR2)	Ccan_OSU1_lncRNA_contig74544.1	Homo sapiens	ENSG00000253200	CCAR2 lncRNA (cell cycle and apoptosis regulator 2)	8.0⨉10^−46^	89	155
AC019068.1; antisense	Ccan_OSU1_lncRNA_contig10709.1	Homo sapiens	ENSG00000233611	AC079135.1 gene, antisense lncRNA (TPA - predicted)	2.4⨉10^−12^	77.6	143
AC083843.1	Ccan_OSU1_lncRNA_contig47288.1	Homo sapiens	ENSG00000253433	AC083843.1 gene, lincRNA (TPA - predicted)	7.7⨉10^−13^	88.4	69
AC095055.1 (antisense to SH3D19)	Ccan_OSU1_lncRNA_contig41532.1	Homo sapiens	ENSG00000270681	SH3D19 antisense noncoding RNA (SH3 domain containing 19)	8.1⨉10^− 58^	82.9	274
AC116667.1; (antisense to ZFHX3)	Ccan_OSU1_lncRNA_contig71613.1	Homo sapiens	ENSG00000271009	ZFHX3 antisense (zinc finger homeobox 3)	1.8⨉10^−47^	83.6	231
AL161747.2; (antisense to SALL2)	Ccan_OSU1_lncRNA_contig44345.1	Homo sapiens	ENSG00000257096	SALL2 lncRNA (spalt-like transcription factor 2)	7.5⨉10^−68^	84.4	288
AP000233.2	Ccan_OSU1_lncRNA_contig22249.1	Homo sapiens	ENSG00000232512	AP000233.2 gene lincRNA (TPA - predicted)	9.0⨉10^−5^	100	31
AP003068.1; (antisense to VPS51)	Ccan_OSU1_lncRNA_contig24716.1	Homo sapiens, Mus musculus, Bos taurus	ENSG00000254501	VPS51 antisense (vacuolar protein sorting 51)	0	93.2	438
AP003068.1; (antisense to VPS51)	Ccan_OSU1_lncRNA_contig55707.1	Mus musculus, Homo sapiens, Gallus gallus	ENSG00000254501	VPS51 antisense/reverse strand (vacuolar protein sorting 51)	1.7⨉10^−83^	92	226
CTA-204B4.6†	Ccan_OSU1_lncRNA_contig29141.1	Homo sapiens	ENSG00000259758	CTA-204B4.6 gene lincRNA (TPA - predicted)	6.2⨉10^− 120^	83.5	491
CTA-204B4.6	Ccan_OSU1_lncRNA_contig30023.1	Homo sapiens	ENSG00000259758	CTA-204B4.6 gene lincRNA (TPA - predicted)	2.1⨉10^− 129^	94.5	308
DNM3OS; (antisense to DNM3)	Ccan_OSU1_lncRNA_contig78034.1	Homo sapiens; various primates	ENSG00000230630	DNM3OS (DNM3 opposite strand/antisense RNA) lncRNA	3.4⨉10^−69^	89.8	216
GNB4; lncRNA isoform*	Ccan_OSU1_lncRNA_contig55083.1	Homo sapiens	ENSG00000114450	GNB4 (guanine nucleotide binding protein (G protein), beta polypeptide 4)	6.4⨉10^−38^	78.8	287
AC007038.2; (antisense to KANSL1L)	Ccan_OSU1_lncRNA_contig54664.1	Homo sapiens, Mus musculus	ENSG00000272807	KANSL1L antisense transcript (KAT8 regulatory NSL complex subunit 1-like)	1.1⨉10^−40^	92	125
KCNA3; noncoding isoform	Ccan_OSU1_lncRNA_contig27553.1	Homo sapiens, Mus musculus	ENSG00000177272	KCNA3 lncRNA (potassium voltage-gated channel, shaker-related subfamily, member 3)	2.3⨉10^− 139^	85.5	502
KCNA3; noncoding isoform	Ccan_OSU1_lncRNA_contig29471.1	Homo sapiens	ENSG00000177272	KCNA3 lncRNA (potassium voltage-gated channel, shaker-related subfamily, member 3)	1.8⨉10^−70^	78.7	475
KCNA3; noncoding isoform	Ccan_OSU1_lncRNA_contig79757.1	Homo sapiens	ENSG00000177272	KCNA3 lncRNA (potassium voltage-gated channel, shaker-related subfamily, member 3)	7.6⨉10^−31^	80.2	197
KCNA3; noncoding isoform	Ccan_OSU1_lncRNA_contig81530.1	Homo sapiens, Mus musculus	ENSG00000177272	KCNA3 lncRNA (potassium voltage-gated channel, shaker-related subfamily, member 3)	7.1⨉10^−61^	87.7	211
LINC01355	Ccan_OSU1_lncRNA_contig54147.1	Homo sapiens	ENSG00000261326	LINC01355 lncRNA	1.0⨉10^− 85^	87.5	295
LMLN; noncoding isoform*	Ccan_OSU1_lncRNA_contig28300.1	Homo sapiens	ENSG00000185621	LMLN (leishmanolysin-like (metallopeptidase M8 family)	3.1⨉10^− 73^	80.4	414
MEG3	Ccan_OSU1_lncRNA_contig11359.1	Homo sapiens, Mus musculus, Pongo abelii	ENSG00000214548	MEG3 lncRNA (maternally expressed 3)	1.6⨉10^− 123^	93	313
MEG3	Ccan_OSU1_lncRNA_contig30419.1	Homo sapiens, Pongo abelii	ENSG00000214548	MEG3 lncRNA (maternally expressed 3)	7.6⨉10^− 124^	93	313
MEG3	Ccan_OSU1_lncRNA_contig6442.1	Homo sapiens, Mus musculus, Pongo abelii	ENSG00000214548	MEG3 lncRNA (maternally expressed 3)	2.2⨉10^−123^	93	313
N4BP2L2-IT2*	Ccan_OSU1_lncRNA_contig81871.1	Homo sapiens	ENSG00000281026	N4BP2L2-IT2 lncRNA (N4BPL2 intronic transcript 2)	2.2⨉10^−6^	76.2	130
NIPBL-DT	Ccan_OSU1_lncRNA_contig25986.1	Homo sapiens	ENSG00000285967	NIPBL lncRNA bidirectional promoter (Nipped-B homolog)	3.6⨉10^−38^	80.9	225
PDK3; noncoding isoform*	Ccan_OSU1_lncRNA_contig72478.1	Homo sapiens	ENSG00000067992	PDK3 (pyruvate dehydrogenase kinase, isozyme 3)	1.8⨉10^−37^	84.2	171
RASSF3; noncoding isoform*	Ccan_OSU1_lncRNA_contig10200.1	Homo sapiens	ENSG00000153179	RASSF3 (Ras associated (RalGDS/AF-6) domain family member 3)	0	83.2	963
RASSF3; noncoding isoform*	Ccan_OSU1_lncRNA_contig10200.2	Homo sapiens	ENSG00000153179	RASSF3 (Ras associated (RalGDS/AF-6) domain family member 3)	0	83.3	962
AC098818.2†; (antisense to BMP2K)	Ccan_OSU1_lncRNA_contig59404.1	Homo sapiens	ENSG00000260278	RP11-109G23.3 gene, antisense lncRNA	4.5⨉10^−59^	83.3	275
TRIM56; sense overlapping	Ccan_OSU1_lncRNA_contig18315.1	Homo sapiens	ENSG00000169871	RP11-395B7.7 gene, sense overlapping lncRNA (TPA - predicted)	4.7⨉10^−28^	72.8	519
RP11-395B7.7	Ccan_OSU1_lncRNA_contig47935.1	Homo sapiens	ENSG00000260336	RP11-395B7.7 gene, sense overlapping lncRNA (TPA - predicted)	9.7⨉10^−22^	73.9	284
AC090948.1	Ccan_OSU1_lncRNA_contig29838.1	Homo sapiens	ENSG00000271964	RP11-415F23.2 gene, antisense lncRNA (TPA - predicted)	1.5⨉10^−26^	93.3	89
AL591848.4†	Ccan_OSU1_lncRNA_contig59344.1	Homo sapiens	ENSG00000260855	RP11-439E19.10 gene, antisense lncRNA (TPA - predicted)	4.9⨉10^−4^	96.9	32
AC022893.2	Ccan_OSU1_lncRNA_contig76877.1	Homo sapiens	ENSG00000260838	RP11-531A24.3 gene, lincRNA (TPA - predicted)	3.6⨉10^−39^	81.4	226
AL355488.1 (antisense to SLC16A4)	Ccan_OSU1_lncRNA_contig17784.1	Homo sapiens	ENSG00000273373	RP5-1074 L1.4 gene, antisense lncRNA (TPA - predicted)	1.0⨉10^−44^	89.9	149
THRB-AS1; (antisense to THRB)	Ccan_OSU1_lncRNA_contig53102.1	Homo sapiens	ENSG00000228791	THRB antisense/reverse strand (thyroid hormone receptor, beta)	6.8⨉10^−18^	80.9	136
TINCR; lncRNA isoform	Ccan_OSU1_lncRNA_contig14850.1	Homo sapiens	ENSG00000223573	TINCR lncRNA (tissue differentiation-inducing non-protein coding RNA)	4.1⨉10^−44^	82.2	225
TUG1; lncRNA isoform	Ccan_OSU1_lncRNA_contig6874.1	Mus musculus	ENSG00000253352	TUG1 lncRNA (taurine upregulated gene 1)	6.2⨉10^−79^	79.9	448
UBR5; lncRNA isoform*	Ccan_OSU1_lncRNA_contig10406.1	Homo sapiens, Bos taurus	ENSG00000104517	UBR5 (ubiquitin protein ligase E3 component n-recognin 5)	0	82.9	977
XIST	Ccan_OSU1_lncRNA_contig185.1	Homo sapiens, Mus musculus	ENSG00000229807	XIST lncRNA (X inactive specific transcript)	3.1⨉10^− 136^	79.7	772

*E*, the *E*-value for the highest-scoring BLASTn match; %ID, percent identity between the contig and matching query sequence, by BLASTn; nt, length of match (nt); *E*-value of “0” means that *E* < 2.23 × 10^− 308^. Columns as follows: “Symbol”, Human Gene Nomenclature Committee gene symbol; “annotation”, classification of the lncRNA transcript type if it is not an obligate lncRNA gene or if it is antisense to a protein-coding gene (*i.* entries with an asterisk after the annotation denote noncoding transcript contigs whose orthologs are *potential* noncoding isoforms; see Methods; *ii.* entries with a dagger after the annotate denote transcripts which have new BLASTn annotations for beaver, as of November 18, 2019); “Contig,”, the name of the transcript contig; “Species”, the species in which orthologs of the contig were detected by sequence similarity; Ensembl Gene ID, the Ensembl gene identifier of the putative human ortholog; “BLASTn annotation”, the annotation of the BLASTn hit corresponding to the statistics in the last three columns (*E*, %ID, nt)

To assess the possible functional coherence of the beaver lncRNAs with known orthologs, we analyzed KEGG biological pathway annotations for the human orthologs of the Table 3 (ortholog-mapped) lncRNAs for statistical enrichment (see Methods). The analysis yielded seven significantly enriched (FDR < 0.05) pathways (Table 4) whose constituent genes are (in human) significantly correlated in expression with the query lncRNAs.

**Table 4:** Results of pathway enrichment analysis of human orthologs of beaver lncRNAs

Pathway name	Gene set size of pathway	Enrichment score (normalized)	FDR adjusted *P-*value
KEGG_RIBOSOME	87	2.48	< 10^− 8^
KEGG_PROTEIN_EXPORT	22	2.38	< 10^−8^
KEGG_NEUROACTIVE_LIGAND_RECEPTOR_INTERACTION	263	1.68	< 10^−8^
KEGG_TASTE_TRANSDUCTION	48	2.20	< 10^−8^
KEGG_REGULATION_OF_ACTIN_CYTOSKELETON	211	1.17	< 10^−8^
KEGG_RNA_POLYMERASE	28	1.91	0.025
KEGG_CALCIUM_SIGNALING_PATHWAY	176	1.86	0.049

The normalized enrichment scores are computed as described in [33]

### Tissue-level expression of beaver lncRNAs

**Fig. 6:**
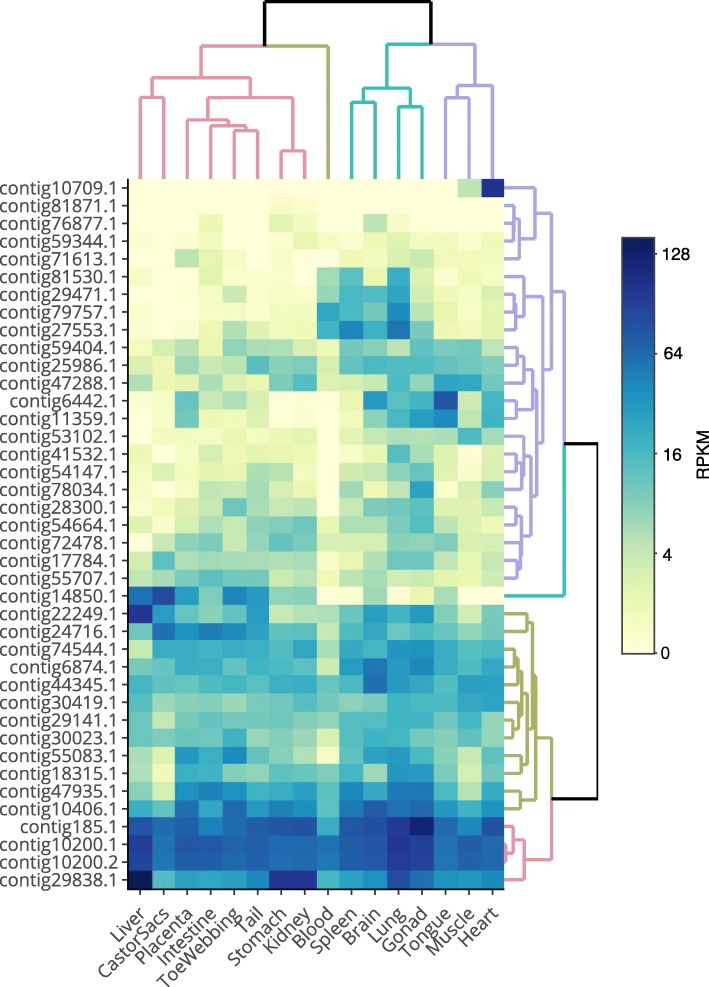
Tissue-specific expression of beaver lncRNAs that are orthologous to known noncoding transcripts. Heatmap rows correspond to the 40 contigs and columns correspond to the 16 tissues that were profiled. Cells are colored by log_2_(1 + RPKM) expression level. Rows and columns are separately ordered by hierarchical agglomerative clustering and cut-based sub-dendrograms are colored (arbitrary color assignment to sub-clusters) as a guide for visualization. Rows are labeled with abbreviated contig names, e.g., contig29838.1 instead of Ccan_OSU1_lncRNA_contig29838.1

Following the lncRNA discovery phase of the analysis, we used RNA-seq to analyze lncRNA levels in the 16 beaver tissues or anatomic structures (the same set of tissues from which we constructed the pooled transcriptome library): whole blood, brain, lung, liver, heart, stomach, intestine, skeletal muscle, kidney, spleen, ovaries, placenta, castor gland, tail skin, toe-webbing, and tongue. For each of the 187 contigs^1^ and in each of the 16 tissues, we estimated the transcript abundance in RPKM (see Additional file 6 Table S2 and Methods). Heatmap visualization of the tissue-specific expression profiles of the 147 novel (Fig. 4) and 40 known (Fig. 6) lncRNA contigs revealed both tissue-specific and ubiquitously expressed beaver lncRNAs.

Among the 147 novel lncRNA contigs, several contigs are notable: contig84039.1 has extremely high (RPKM 1910) expression in castor sac relative to the other tissues (average RPKM of 64); contig81051.1 was ubiquitously expressed and had overall highest expression (average RPKM of 433); and a cluster of four contigs (contig80136.1, contig83384.1, contig72740.1, and contig 83,657.1) are specifically expressed in stomach and kidney. From a tissue lncRNA expression standpoint, kidney and stomach clustered together in both the known and novel lncRNA datasets, consistent with previous findings from tissue transcriptome analysis [34]. Brain tissue was notable for having several tissue-specific lncRNA contigs (contig76717.1, contig65642.1, and contig43610.1). Finally, the heatmap analysis revealed that contig44966.1 is strongly expressed (over 20 RPKM) in spleen and ovary (annotated as “gonad”), but not in other tissues (Fig. 4, left panel, fifth row from bottom); it has no matches in the NCBI non-redundant nucleotide database, lncRNAdb [35], or in RNA Central [36], suggesting that if it is indeed a functional beaver lncRNA, it is not known to be conserved in other rodents.

As an independent check on the biological validity of the RNA-seq-based lncRNA gene expression measurements, we compared the log_2_ expression in muscle of all 187 known and novel lncRNAs as measured in our study and by the Lok et al. study [7], which were obtained using different sequencing technologies and using tissue samples from different beavers. We found that the two sets of lncRNA expression measurements were correlated at *R* = 0.66 (*P* < 10^− 15^), as shown in Additional file 2 Figure S2.

### Gene correlation analysis of novel lncRNA contig81051.1

We selected one putative novel lncRNA contig with very high overall expression level, contig81051.1, to explore its possible downstream regulated genes using coexpression analysis. We mapped ten potential target genes by identifying mRNA transcript contigs whose RNA-seq expression levels across the 16 beaver tissues were correlated with contig81051.1 at *R* > 0.94. We were able to map eight of the genes to mammalian orthologs (*ERGIC2*, *RAD23*, *TP53RK*, *SCRN3*, *RAD21*, *RAD5*, *SECISBP2*, *PPARD*) (see Methods). The functional annotations of the eight ortholog genes are enriched for the Gene Ontology biological process *DNA Recombination* (*P* = 0.000213), suggesting that the lncRNA contig81051.1 may be involved in regulating chromatin maintenance.

For the 40 beaver lncRNA contigs with known orthologs (Fig. 6), four notable tissue-level expression patterns emerged. First, expression of contig10709.1, whose human ortholog *AC079135.1* is an antisense lncRNA to the human gene *Ankyrin repeat and SOCS box containing 18* (*ASB18*), was specific to heart and skeletal muscle, consistent with human *ASB18* which is expressed in heart and skeletal muscle, according to the Human Protein Atlas (HPA) [37]. Second, contigs contig6442.1 and contig11359.1, which are orthologs of the mammalian lncRNA *MEG3*, are strongly expressed in placenta, spleen, brain, ovary, tongue, lung, and heart; the human ortholog is strongly expressed (at least 10 tags per million) in brain, ovary, spleen, lung, and heart according to data from the genotype tissue-expression (GTEx) project [38]. For contig29838.1, a ubiquitously expressed antisense lncRNA with specifically high expression in liver (RPKM of 1149), the human ortholog antisense lncRNA *RP11-415F23.2*, is expressed in liver and endothelial cells, according to the ANGIOGENES database [39]; moreover, the human antisense lncRNA’s neighboring gene, *Raftlin, lipid raft linker 1* (*RFTN1*), is strongly expressed in liver, stomach, kidney, and ovaries, according to the HPA. Finally, we note that four beaver lncRNAs (contig81530.1, contig29471.1, contig79757.1, and contig27553.1) all cluster together in terms of gene expression and they are all orthologous to noncoding isoforms of the human gene *potassium voltage-gated channel, shaker-related subfamily, member 3* (*KCNA3*); the four beaver lncRNAs are expressed in blood, spleen, brain, and lung, as is human *KCNA3*, according to the HPA.

For the lncRNA contigs with known orthologs that are expressed in all of the beaver tissues, in general their human orthologs are ubiquitously expressed. For example, contig185.1, whose expression level varies from 65 to 843 RPKM in the beaver tissues, is orthologous to *XIST*, which is ubiquitously expressed in human tissues according to GTEx. Similarly, contig10200.1 and contig10200.2 are expressed in the range of 92–476 RPKM in beaver tissues, and their human ortholog (*RASSF3*) is ubiquitously expressed in the 33 human tissue types profiled by the GTEx project. Finally, contig10406.1 is ubiquitously expressed in beaver with lowest expression in whole blood and castor sacs; its human ortholog, *UBR5*, also is ubiquitously expressed with low expression in blood, according to GTEx.

### Secondary structure analysis

**Fig. 7:**
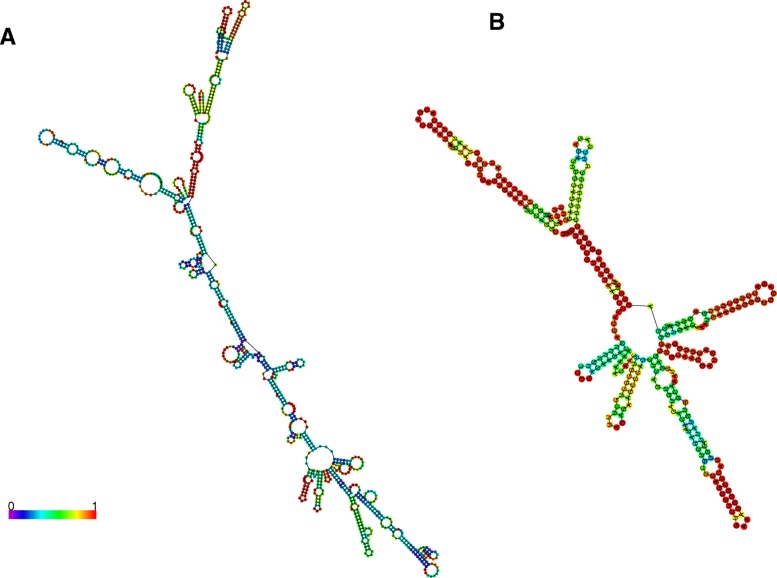
Predicted minimum-free energy secondary structures of the putative beaver *MEG3* lncRNA Ccan_OSU1_lncRNA_contig11359.1 (**a**) and the homologous sequence of human *MEG3* (**b**). False color indicates pairing probability (see colormap in panel A)

**Fig. 8:**
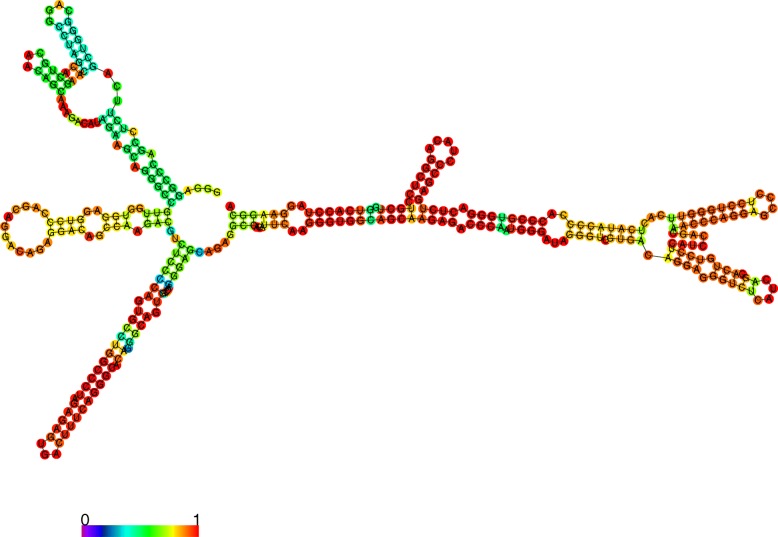
Predicted minimum-free energy secondary structure of the novel spleen- and ovary-specific lncRNA Ccan_OSU1_lncRNA_contig44966.1, showing relatively high pairing probabilities. False color indicates base pairing probability (see colormap)

We selected two lncRNA contigs, a “known” lncRNA (Ccan_OSU1_lncRNA_contig11359.1, a putative beaver ortholog of human lncRNA *MEG3*) and a novel lncRNA (Ccan_OSU1_lncRNA_contig44966.1, whose expression is ovary- and spleen-specific) to analyze from the standpoint of computationally predicted secondary structure (see Methods). The minimum-free energy secondary structure of the putative beaver *MEG3* lncRNA (Fig. 7a) has a three-branched structure that is strikingly similar to a previously published secondary structure for human *MEG3* [40] (Fig. 7b), with the three motif domains clearly evident. Furthermore, we confirmed the orthology of Ccan_OSU1_lncRNA_contig11359.1 to the lncRNA MEG3 using the tool Infernal to align its secondary structure to the Rfam *MEG3* motif (see Methods), with average per-base alignment probability of 0.959. The spleen- and ovary-specific lncRNA Ccan_OSU1_lncRNA_contig44966.1 has the lowest MFE of any novel contig (see Fig. 1a) and a relatively high-confidence secondary structure—with four branches from a central bubble—based on its base pairing probability (Fig. 8). Because interspecies conservation of lncRNAs is reported to be lower at the sequence level than at the level of secondary structure, we used a *k*-mer based tool (SEEKR [41]; see Methods) for assessing whether Ccan_OSU1_lncRNA_contig44966.1 has any orthologs in the Mouse GENCODE lncRNA set of transcripts [42]. The highest correlation coefficient was 0.61, with the highest-scoring lncRNA (*Gm9754–201*) showing little structural similarity. The analysis revealed no evidence of the existence of a murine ortholog of Ccan_OSU1_lncRNA_contig44966.1.

## Discussion

Although this work focused on discovering beaver lncRNAs using multi-tissue transcriptome profiling, some novel aspects of the bioinformatics workflow that we used are worth noting. Previous lncRNA discovery approaches have substantially leveraged an annotated reference genome and/or transcriptome [27, 43–47]. In contrast, because no consensus beaver transcriptome existed, the foundation for our approach was de novo transcriptome assembly. Thus, our approach is applicable to the case of a draft reference genome with only computationally generated annotations, or even to an organism where no reference genome assembly is available. We also systematically curated beaver lncRNA contigs that had detectable orthologs in order to determine if the orthology was to a known lncRNA or to an obligate noncoding transcript-specific isoform of a protein-coding gene; this Ensembl-based disambiguation of orthology relationships is, so far as we know, unique in lncRNA profiling studies. Despite having termed the 40 contigs with known orthologs as “known lncRNA” to distinguish them from the 147 contigs with no detectable orthologs, we note that the 40 “known” lncRNAs are also new insofar as they have been identified (and their tissue expression distributions mapped) in beaver for the first time.

The sixteen beaver tissues that we profiled constitute a broad transcriptome atlas that extends beyond the three beaver tissues previously profiled [7]. While other beaver tissues (e.g., testis) remain to be profiled in a future study, sequence alignment of a set of 4104 high-confidence pan-vertebrate genes (BUSCO genes) vs. a concatenation of six beaver transcript assemblies that we generated using four assemblers indicates that at least 91% of mammalian BUSCO genes have beaver orthologs.

In our compendium of beaver lncRNAs, one distinction between known and novel contigs is worthy of discussion: contigs with known orthologs were on average ~ 2.5-fold longer than novel lncRNAs (Fig. 2). Given the likelihood that many if not most of the novel contigs are partial transcripts, it seems plausible that this difference in lengths reflects the fact that a longer contig is less likely to miss the phylogenetically conserved portion of the gene. Nevertheless, it is worth considering whether an evolutionary argument explains the discrepancy, namely, that evolutionarily more ancient lncRNA genes tend to be longer, as has been reported for protein-coding genes [48].

Genomic analysis suggests biological relevance for the 147 novel lncRNA contigs yielded by our study. When we mapped the lncRNAs to the Oregon State University draft genome assembly (see Methods), 82.3% of our novel contigs mapped to the genome with an alignment length that was in excess of 90% of the contig’s length. This suggests that the contigs are properly assembled and (together with the RPKM values) suggests that they are transcribed from the beaver genome. Furthermore, the mapping serves as a preliminary step in examining the genomic context of the putative lncRNA gene; confirming placement between a transcriptional start site and transcriptional end site would be a next step in confirming or rejecting the putative novel lncRNAs. The finding of several brain-specific lncRNAs is consistent with findings from the human GENCODE study that a large fraction of tissue-specific lncRNAs are expressed in brain [26]. Finally, the pathway enrichment analysis of human orthologs of the 40 ortholog-mappable lncRNA contigs (which are biased toward high expression in at least one tissue type) identified several pathways, including “ribosome”, “calcium signaling”, “protein export”, and “neuroactive ligand-receptor interaction”. A signature adaptation of the beaver is its ability to withstand hypoxia, the response to which in mammals is known to reprogram intracellular calcium signaling [49], downregulate protein synthesis [50], and activate neuroendocrine [51] pathways.

One caveat of this analysis is that, in light of a recent report that some lncRNAs may encode micropeptides [52, 53], the stringent cutoff used to filter for coding potential of the lncRNA contigs likely eliminated some lncRNA contigs. This reduction in sensitivity is a trade-off for controlling the rate of false positive identifications of protein-coding transcripts as lncRNAs. Further improvements in the sensitivity of bioinformatic methods for scoring coding potential are needed in order to enable more comprehensive discovery of lncRNAs while maintaining stringent control of false positives. Relatedly, although (as described above) various lines of evidence suggest that the 187 contigs are lncRNAs, targeted and replicated validation experiments would be required in order to conclusively demonstrate their expression in beaver tissues.

The tissue-specific analysis of beaver lncRNAs yielded both novel findings and supporting evidence for function. For several of the 40 lncRNA contigs with known ortholog genes (e.g., *MEG3, RP11-415F23.2, AC079135.1, KCNA3*), we found consistent patterns of tissue-specific expression between the beaver transcript contigs and the ortholog genes, bolstering evidence for the ortholog mappings and confirming previous reports that tissue-specific expression of noncoding RNAs is often phylogenetically conserved across ortholog pairs [54]. The finding that the proportion of lncRNA contigs with known orthologs whose orthologs are antisense transcripts is relatively high (12 out of 40) is consistent with the GENCODE study’s finding that a high proportion of human genic lncRNA transcripts are antisense [26]. For *MEG3*, the consistency of predicted secondary structure of the beaver lncRNA contig and the published *MEG3* Rfam motif is highly suggestive of a correct annotation. Our finding of a spleen- and ovary-specific novel lncRNA, Ccan_OSU1_lncRNA_contig44966.1, is certainly plausible given previous published work systematically identifying ovary-specific lncRNAs in pigs [28]; given the high pairing probabilities in the MFE secondary structure of that lncRNA, Ccan_OSU1_lncRNA_contig44966.1 would be a strong candidate for targeted studies to ascertain its function in beaver. More broadly, the overall pattern of tissue-specific expression of the known lncRNA contigs in beaver grouped related tissues (e.g., skeletal muscle, heart, and tongue in one subgroup, and kidney and stomach in another subgroup), consistent with previously published results for mouse [34]. The finding of probable beaver orthologs of noncoding isoforms of protein coding genes–with consistent patterns of tissue expression–is consistent with previous reports that lncRNAs and nearby protein-coding genes are often correlated in terms of tissue-specific expression [55]; it is also consistent with previous estimates that up to 68% of genes can encode noncoding isoforms [55].

## Conclusions

Via transcriptome profiling of sixteen tissues in the American beaver, we identified 40 known lncRNAs and 147 potential novel lncRNAs and we profiled their expression levels in sixteen tissues in a female adult beaver. We annotated the 40 known lncRNAs based on their orthologs and confirmed consistency of tissue expression (between beaver and the orthologous species) for several of the lncRNAs for which ortholog tissue expression data could be obtained. Eight of the novel lncRNA contigs have especially strong evidence across five different heuristics for biological significance and may be the most promising contigs to use as a basis for hypothesis generation for targeted functional investigations. The analysis workflow that we used is general with respect to the species and could be used for RNA-seq-based lncRNA discovery in other species. To the best of our knowledge, this work is the first comprehensive tissue transcriptome analysis of the beaver. The sequence data resulting from this analysis (which are deposited in a public repository; see Availability of data and materials) will provide a foundation for improving annotation of the beaver genome, characterizing tissue expression of all beaver genes, extending rodent comparative genomics, and elucidating the biological mechanisms underlying the beaver’s unique adaptations.

## Methods

### Sample collection

From a donated cadaver of a euthanized pregnant female beaver, we collected sixteen tissues: whole blood, brain, lung, liver, heart, stomach, intestine, skeletal muscle, kidney, spleen, ovaries, placenta, castor gland, tail, toe-webbing, and tongue. We stabilized blood (200 μL), liver (four 11 mm^3^ cubes), and brain (four 24 mm^3^ cubes) samples in 600 μL TRI reagent (Zymo Research, Irvine, CA) per tissue type and stored them at − 80 °C. We stabilized four 20 mm^3^ cubes each from the other solid tissue types (excluding liver and brain) in 1 mL RNAlater (Qiagen, Hilden, Germany). Additionally, from a male beaver called ‘Filbert’ (four years of age) at the Oregon Zoo that was anesthetized (for a routine medical examination on August 18, 2015) by inhaled isoflurane, 2 mL of peripheral blood was obtained by tail venipuncture for transcriptome and genome sequencing (this was the beaver whose DNA was sequenced for the Oregon State University beaver genome assembly).

### RNA isolation

For solid tissues that were preserved in RNAlater, we removed the tissue sample from the RNAlater reagent and snap-froze the tissue block in liquid nitrogen, ground it with mortar-and-pestle, and homogenized the tissue in 600 μL TRI Reagent. From each of the 16 homogenized tissue samples, we isolated total RNA using the Zymo Direct-zol RNA MiniPrep (Zymo Research) kit. For all tissues, we obtained RNA Integrity Number (RIN) quality scores using an Agilent Bioanalyzer (Agilent Technologies, Santa Clara, CA); all RIN scores were above 6.2.

### Sequencing

*All-tissues pooled-RNA (“pan-tissue”) transcriptome profiling:* From pooled polyadenylated RNA from all tissues (equal amounts from each tissue), we prepared a strand-specific RNA-seq library for Illumina sequencing using the PrepX RNA-Seq for Illumina Library Kit (WaferGen Biosystems, Fremont, CA). We sequenced the pooled polyA+ transcriptome library (WaferGen Biosystems) on one lane of an Illumina MiSeq 3000 (Illumina, San Diego), obtaining approximately 3 million read pairs (2 × 76 sequencing cycles).

*Tissue transcriptome atlas:* For each of the sixteen tissues, we prepared barcoded cDNA libraries for paired-end Illumina sequencing in triplicate using the Truseq Stranded mRNA Library Prep Kit (Illumina). We sequenced the sixteen tissue samples for 2 × 150 cycles on one lane of the HiSeq 3000 (Illumina), obtaining an average of 21.4 million read pairs per sample (across-samples standard deviation of 3.0 million read pairs).

### Gene prediction and genome annotation

For the reference beaver genome, we used the Oregon State University draft beaver genome [6] assembly (the bgp_v1 assembly; see Declarations: Availability of data and materials). We generated a repeat-masked version of the genome assembly using RepeatMasker [56] with the GIRI rodentia repeat database [57]. We generated gene predictions and genome annotations using three different tools: GeneMark.hmm [58] (with de novo model training); SNAP [59] using the provided mam54 model; and MAKER v.2.31 [60], with the latter incorporating both the bgp_v1 assembly and the RNA-seq data from the beaver blood sample that was obtained by tail venipuncture (see Sample Collection). Additionally, as input to MAKER for genome annotation, we used the following supplementary files: ESTs (this file was generated by running TransDecoder (github.com/TransDecoder) on the all-tissues transcriptome assembly), and protein sequences for six other species from the Order Rodentia (*Cavia porcellus*, *Oryctolagus cuniculus*, *Rattus norvegicus*, *Ictidomys tridecemlineatus*, *Dipodomys ordii*, *Mus musculus*) obtained from Ensembl [6] Release 87.

### Pan-tissue Transcriptome assembly

Starting with the paired FASTQ files from the MiSeq sequencing of the pooled tissue RNA libraries, we bioinformatically trimmed overrepresented polyadenine and adapter sequences using fastq_clipper v534 (github.com/ExpressionAnalysis/ea-utils). The FASTQC [61] sequence quality report showed per-base median PHRED scores exceeding 30 for all cycles. We screened the trimmed reads for contamination using NCBI BLASTn [62] against the NCBI nucleotide (nt) database and found no evidence of contamination. We generated a de novo transcriptome assembly using the trimmed reads as input to Trinity [63]. We then used Transfuse v0.5.0 [64] with the default *i* value of 1.0 and the Trinity assembly as input to generate a non-redundant transcriptome. This step also had the effect of reducing computational complexity for the remainder of the pipeline.

To estimate the transcriptome coverage of highly-conserved mammalian genes across the sixteen tissues, we used the BUSCO software v2.0 [24] on six pan-tissue transcriptome assemblies: (i) the de novo Trinity assembly, before modification by transfuse, (ii) a transcript file generated using Maker Gene Models [60] analysis of the reference genome; (iii) transcript files from de novo assemblies (of the tissue RNA-pooled RNA-seq data) that we generated using Velvet-Oases [65] and BinPacker [66], (iv) a transcript file that we generated via a reference-guided assembly using the Trinity assembler, and (v) another de novo Trinity assembly in which orphan reads (whose paired-end partner read had been eliminated during quality assessment) had been eliminated from the input data. BUSCO was run with lineage dataset mammalia_odb9, mode transcriptome, species human, and *E*-value cutoff of 10^− 3^. The six assemblies were analyzed individually and as a single concatenated assembly.

### Novel lncRNA discovery pipeline

**Fig. 9:**
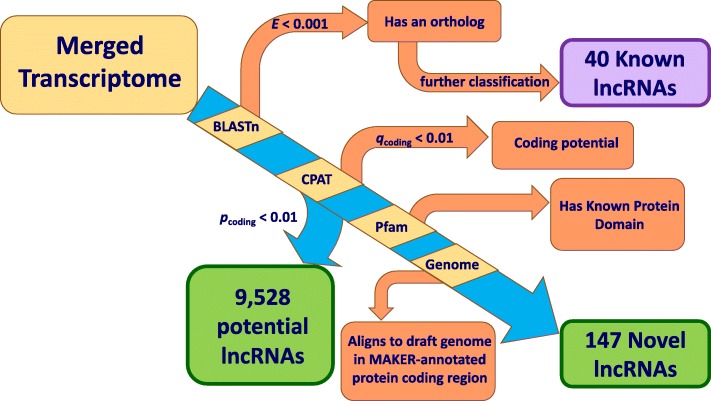
Overview of the computational pipeline for identifying beaver lncRNAs. Transcript contigs from the consensus transcriptome (“Merged Transcriptome” above) were sequentially filtered using (1) Basic Local Alignment Search Tool for nucleotide sequence (BLASTn) against the NCBI nucleotide database to eliminate probable orthologs of protein-coding genes, known lncRNAs, and other non-lncRNA transcript types; (2) CPAT to detect and eliminate contigs with protein-coding ORFs or nucleotide hexamer usage patterns that are consistent with protein coding genes; (3) HMMscan scan against the Pfam database to identify matches to protein domain motifs; and (4) BLASTn alignment against the OSU draft beaver genome assembly and eliminating those contigs that overlapped with scaffold regions that were annotated (by MAKER) as protein-coding genes. Contigs discovered by the annotation pipeline that are orthologs of known lncRNAs are shown in purple, and novel noncoding contigs identified by the annotation pipeline are shown in green

We used a multi-step process to filter the merged transcriptome assembly to eliminate contigs that had evidence for coding potential or that had been studied before in an orthologous system (Fig. 9). Since all contigs in the merged assembly were at least 200 nt long (the generally accepted minimum length for a lncRNA [1]), it was not necessary to filter contigs for minimum nucleotide length. For each of the 86,714 contigs, we searched for orthologs using BLASTn [67] against the NCBI Nucleotide Database [68], with an *E*-value threshold of 10^− 3^. We classified each contig by its BLASTn results into one of three groups: (1) the contig has a significant BLASTn match to a protein-coding gene or non-lncRNA transcript (e.g., rRNA) in another species [such contigs were excluded from further analysis]; (2) the contig has at least one significant BLASTn match to a noncoding transcript in another species [we found 113 such contigs, and we manually filtered and curated them as described below in Sec. “BLASTn-based classification of noncoding transcript contigs”]; and (3) the contig did not have a significant BLASTn match [for these, we checked for coding potential as described below].

There were 32,309 “no orthologs” contigs in group (3); for each of them, we quantified the potential for coding using CPAT [20], as follows. For each contig, from the CPAT coding probability score *p*, we computed an adjusted coding probability *q* to account for multiple hypothesis tests. We generated a set of 9528 “probable noncoding” contigs for which *p* < 0.01, whose sequences are provided as Additional file 3 Supplementary Data 1. To obtain a more stringent set of noncoding contigs for downstream analysis, we filtered by CPAT score for “high-confidence noncoding” contigs for which *q* < 0.01 (corresponding to Benjamini-Hochberg [69] false discovery rate (FDR) < 0.01), yielding a set of 182 contigs. We analyzed the 182 high-confidence noncoding, “no orthologs” contigs using the software tool HMMscan [20, 70] to identify matches to sequence patterns annotated for known protein domains from the Pfam database [71], similar to previous RNA-seq-based screens for lncRNAs [72, 73]. For this analysis we used the HMMscan database-defined significance thresholds (“gathering threshold” option), with a match being grounds for excluding a contig (consistent with a previous report that the vast majority of bioinformatically predicted lncRNAs do not contain Pfam matches [53]).

In order to eliminate contigs that are likely untranslated region (UTR) portions of protein-coding transcripts, we aligned the remaining 182 high-confidence noncoding, “no orthologs” contigs to scaffold sequences of the Oregon State University draft beaver genome assembly using BLASTn. Those transcript contigs that matched within a genome region that was annotated by MAKER as a protein-coding gene were dropped from further consideration (see Table 5 for the specific types of MAKER annotations that we used for identifying probable protein-coding mRNA contigs for exclusion from the analysis). A total of 147 contigs passed successfully through all these filters and therefore were classified as putatively novel (as in “no known orthologs”) lncRNAs.

**Table 5:** Evidentiary criteria for filtering transcript contigs based on the MAKER gene annotation features

	Annotation Tool	Annotation Call
Basis for Exclusion as lncRNA	blastx	protein_match
	genemark	match, match_part
	maker	CDS
	protein2genome	match_part, protein_match
	snap_masked	match, match_part
	tblastx	match_part, translated_nucleotide_match
not basis for Exclusion as lncRNA	blastn	expressed_sequence_match; match_part
	blastx	match_part
	cdna2genome	expressed_sequence_match; match_part
	est2genome	expressed_sequence_match; match_part
	maker	exon, gene, mRNA
	repeatmasker	match; match_part

### BLASTn-based classification of noncoding transcript contigs

In order to filter the contigs that had at least one significant (*E* < 10^−3^) BLASTn match to a noncoding transcript in another species (“known lncRNA”; see Sec. “Novel lncRNA Discovery Pipeline”) and to eliminate ones that could be explained as noncoding portions of orthologous protein-coding genes, we classified the “ortholog of known lncRNA” contigs based on their BLASTn hits profile, into three groups: “probable lncRNA”, “possible lncRNA”, or “unlikely to be lncRNA” (Additional file 5 Table S1). Contigs in the last category were excluded from further analysis. We classified contigs based on their BLASTn matches, as follows:

1. We ignored a match if any of the following phrases (or their abbreviations) appeared in the subject sequence title: predicted, synthetic construct, bacterial artificial chromosome, P1-derived artificial chromosome, predicted gene, transgenic, mutant allele, clone, cloning vector, hypothetical, complete genome.
2. If a match was to a Third Party Annotation (TPA) transcript sequence, we retained the match *only* if the query and subject sequences aligned with consistent orientation for the strand information for the TPA record (i.e., sense or antisense strand).
3. We classified each contig based on the remaining (after applying filters 1 and 2) BLASTn matches, as (i) *probable lncRNA* if only matches to *known* lncRNA transcripts in other species remained or if the matches to known lncRNA transcripts outnumbered *and* were higher in percent identity than any matches to protein-coding transcripts; (ii) *possible lncRNA* if both lncRNA and protein-coding mRNA BLASTn matches were approximately equally abundant and of approximately equal quality as measured by length and percent identity of the BLASTn hit; or (iii) *unlikely to be lncRNA* if there were more than ten BLASTn matches with less than 20% of them to a known lncRNA (unless the lncRNA matches were consistent across species; also see Step 4).
4. We annotated any contigs that did not fall into the above classification categories based on manual inspection of the Ensembl gene model in the context of the contig’s Basic Local Alignment Tool (BLAT) match to the human (GRCh38) or mouse (GRCm38) genome assemblies. A BLAT match of a contig to a noncoding exon that is annotated as present *only in lncRNA isoform(s)* of a protein-coding gene was taken as sufficient evidence to annotate the contig as a probable lncRNA.

This annotation pipeline identified 40 contigs (Fig. 9, purple rectangle) that are orthologs of known lncRNAs or noncoding isoforms of protein-coding genes.

### Validation analysis

To validate the 147 high-confidence lncRNA contigs, we aligned them against an independently-generated beaver genome assembly [7] that was generated using a different blood sample, a different sequencing technology (PacBio SMRT DNA sequencing) and a different assembly tool (Canu) than were used to obtain the OSU beaver genome assembly. We obtained the sequence file Castor_canadensis.C.can_genome_v1.0.dna.nonchromosomal.fa from Ensembl and aligned the 147 contigs against it using BLASTn with default parameters. For the secondary analysis of skeletal muscle RNA-seq data from the Lok et al. study [7], we downloaded the SRA archive SRR5149357, extracted FASTQ data using SRA-toolkit fastq-dump 2.9.6, aligned reads to the FASTA-format lncRNA contig assembly using BWA MEM, and counted aligned reads for each contig using samtools idxstats.

### Contig analysis

We calculated the average depth of coverage for the 40 known and 147 novel noncoding transcript contigs using the formula Coverage = (# reads mapped to the contig) × (read length) / (contig length). For assessing consistency of transcript contigs with the reference genome, we aligned novel lncRNA contigs to the beaver reference genome scaffolds using bwa mem [74] (v0.7.15) with the default settings. We computed average contig coverage of the contigs by the RNA-seq reads, using samtools v1.9.

### Tissue atlas of lncRNAs

For the tissue-specific RNA-seq profiling, starting with FASTQ files, we trimmed adapters using cutadapt [75] v1.8.1, aligned to the multipart FASTA file of contig sequences for all 187 candidate lncRNAs using BWA MEM, and counted reads on a per-contig basis using samtools v1.4 [76]. For each contig and each tissue, we computed RPKM values as follows:

$$ \mathrm{RPKM}=\frac{2\times \left(\#\mathrm{reads}\ \mathrm{mapped}\ \mathrm{to}\ \mathrm{contig}\right)}{\left(\mathrm{length}\ \mathrm{of}\ \mathrm{contig}\right)\times \left(\#\mathrm{total}\ \mathrm{RNA}\ \mathrm{reads}\ \mathrm{in}\ \mathrm{tissue}\right)}\times {10}^9. $$

### Secondary structure analysis for specific lncRNAs of interest

We computed the Minimum Free Energy (MFE) for all contigs using the command-line version of the RNAfold structure prediction software [77] v2.2.5. For two specific lncRNA contigs of interest (Ccan_OSU1_lncRNA_contig11539.1 and Ccan_OSU1_lncRNA_contig44966.1), we obtained secondary structure diagrams and secondary structural information using the tool RNAfold WebServer (rna.tbi.univie.ac.at), which is based on the ViennaRNA v1.8.5, with the default settings. For *k*-mer based orthology analysis, we used the SEEKR web tool (seekr.org) using *k* = 4 and specifying the “All Mouse lncRNA” set for comparison and normalization. We tested the sequence for contig Ccan_OSU1_lncRNA_contig11539.1 for secondary structure-based orthology against a *MEG3* motif model (accession RF01872 in the RNA motif database, Rfam [78]) using Infernal v1.1.2 [79].

### Pathway enrichment analysis of lncRNAs with known orthologs

We mapped the 40 beaver lncRNA contigs with known human orthologs to 31 human Ensembl gene IDs. For the 31 Ensembl genes, we analyzed biological pathway annotations from the Kyoto Encyclopedia of Genes and Genomes (KEGG) [80] for enrichment using the R software package LncPath (version 1.1) [81], which uses Gene Set Enrichment Analysis [33]. We filtered the pathways for significant enrichment using a false discovery rate cutoff of 0.05.

### Functional enrichment analysis of coexpressed genes

We aligned the tissue-specific RNA-seq reads to the Trinity de novo transcriptome assembly (see Methods) contigs using bwa mem with default parameters. For each tissue sample, we obtained counts of aligned reads for each Trinity transcriptome contig using the idxstats command from samtools. For each tissue sample, we normalized read counts by the total number of reads in the sample and computed the log_2_ of the zero-inflated normalized counts. For each Trinity transcript contig, we computed the Pearson correlation coefficient of its log_2_ RNA-seq counts with the log_2_ RNA-seq counts for contig81051.1. We used NCBI BLASTn for ortholog mapping and Enrichr for the functional enrichment analysis of the orthologs of co-expressed genes.

## Supplementary information

**Additional file 1: Figure S1.** Gapped genome alignment length of novel lncRNA contigs, as a percentage of contig length**.** The percentage can be over 100% because the gapped alignment allows intervening unpaired bases in either sequence (transcript contig or draft genome scaffold)

**Additional file 2: Figure S2.** Skeletal muscle lncRNA expression is consistent between beavers. Skeletal muscle gene expression of each of 187 known and novel lncRNA contigs in the present study and in the Lok et al. study [7]. Each mark corresponds to a single lncRNA contig

**Additional file 3: **Supplementary Data 1. [SuppData1.txt] Text file of 9528 potential lncRNA transcript contigs, in FASTA format. These contigs have no known orthologs (by BLASTn) and low coding potential scores (CPAT *p* < 0.01)

**Additional file 4: **Supplementary Data 2. [SuppData2.txt] Text file of 187 known and novel lncRNA transcript contigs, in FASTA format*.* The 147 novel contigs have no known orthologs (by BLASTn), very low coding potential scores (CPAT *q* < 0.01), and no Pfam domain matches. The 40 known lncRNA transcript contigs are listed first, followed by the novel contigs

**Additional file 5: Table S1.** Manual curation of the 40 known lncRNAs*.* Columns are as follows: Contig, the query contig’s identifier; Category, the classification of the contig (“probable lncRNA” or “possible lncRNA”, as per Methods Sec. “BLASTn-based classification of noncoding transcript contigs”); Matching Sequence (subject), the subject sequence identifier(s) as per BLASTn; Species, the species of the subject from the previous column; Description, the BLASTn descriptor of the subject sequence; E-value, BLASTn provided E-value for the query-subject pair; %ID, BLASTn provided percent identity between the contig and matching query sequence; length of match, BLASTn provided length, in nucleotides, of query-subject alignment. Only those subject-query pairs that were retained after steps 1 and 2 of curation, as described in the Methods Section, “BLASTn-based classification of noncoding transcript contigs”, are included in the table. Contigs are listed in ascending numerical order

**Additional file 6: Table S2.** Tissue-specific RPKM per contig*.* Columns are as follows: Contig, the contig’s identifier; type, the classification of the contig as “known” or “novel”; remaining columns are of the format “RPKM_TissueType”, where TissueType is one of the 16 tissues collected and profiled (see Methods Section “Sample Collection”). Values in these columns are the tissue-specific RPKM for the contig, calculated as described in the Methods Section “Tissue Atlas of lncRNAs”. Known lncRNA transcript contigs are listed first, followed by novel, with contigs listed in ascending numerical order within each category

## Data Availability

The beaver reference genome FASTQ files are available under NCBI BioProject Accession PRJEB19765 and the genome assembly under GenBank Accession GCA_900168385.1. The pooled RNA-derived FASTQ files are available under EBI ArrayExpress accession number E-MTAB-6258. The tissue-specific RNA-derived FASTQ files are available under EBI ArrayExpress accession number E-MTAB-8038.

## Abbreviations

BLASTn: Basic Local Alignment Search Tool for nucleotide sequence
BLAT: Basic Local Alignment Tool
BUSCO: Benchmarking Universal Single-Copy Ortholog
CPAT: Coding Potential Assessment Tool
LncRNA: Long noncoding RNA
MFE: Minimum free energy
ORF: Open reading frame
RPKM: Reads Per Kilobase of transcript per Million
RRNA: Ribosomal RNA

## Footnotes

1 In this subsection, in the interest of brevity, we identify contigs without the “Ccan_OSU1_lncRNA_” prefix.
