# Supplementary figures and images for: Pan-tissue transcriptome analysis of long noncoding RNAs in the American beaver Castor canadensis
Source: BMC Genomics. 2020 Feb 12;21:153. doi: 10.1186/s12864-019-6432-4 (PMC7014947; doi:10.1186/s12864-019-6432-4)

Density

40 60 80 100

Alignment Length as Percent of Contig Length

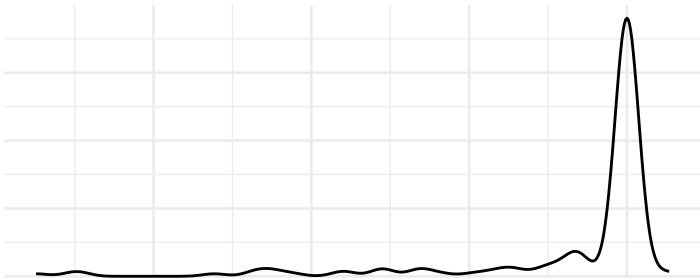

Supplement: Supplementary file 1 — Additional file 1: Figure S1. Gapped genome alignment length of novel lncRNA contigs, as a percentage of contig length. The percentage can be over 100% because the gapped alignment allows intervening unpaired bases in either sequence (transcript contig or draft genome scaffold) [file 12864_2019_6432_MOESM1_ESM.pdf]

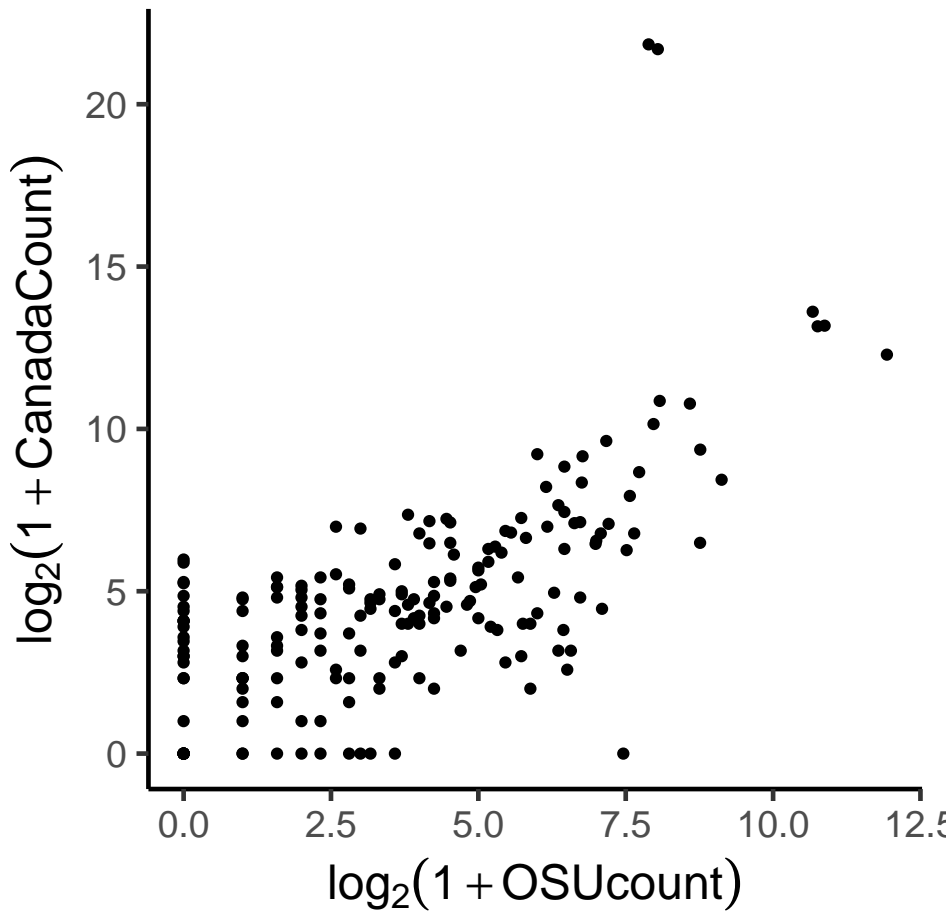

Supplement: Supplementary file 2 — Additional file 2: Figure S2. Skeletal muscle lncRNA expression is consistent between beavers. Skeletal muscle gene expression of each of 187 known and novel lncRNA contigs in the present study and in the Lok et al. study [7]. Each mark corresponds to a single lncRNA contig [file 12864_2019_6432_MOESM2_ESM.pdf]
